# *Bacillus subtilis* Expressing the Infectious Pancreatic Necrosis Virus VP2 Protein Retains Its Immunostimulatory Properties and Induces a Specific Antibody Response

**DOI:** 10.3389/fimmu.2022.888311

**Published:** 2022-06-01

**Authors:** Félix Docando, Noelia Nuñez-Ortiz, Gabriela Gonçalves, Cláudia R. Serra, Eduardo Gomez-Casado, Diana Martín, Beatriz Abós, Aires Oliva-Teles, Carolina Tafalla, Patricia Díaz-Rosales

**Affiliations:** ^1^ Fish Immunology and Pathology Group, Animal Health Research Centre (CISA), National Agricultural and Food Research and Technology Institute (INIA), Spanish National Research Council (CSIC), Madrid, Spain; ^2^ Universidad Autónoma de Madrid, Madrid, Spain; ^3^ Centro Interdisciplinar de Investigação Marinha e Ambiental (CIIMAR), Universidade do Porto, Terminal de Cruzeiros do Porto de Leixões, Matosinhos, Portugal; ^4^ Departamento de Biologia, Faculdade de Ciências, Universidade do Porto, Rua do Campo Alegre, Porto, Portugal; ^5^ Department of Biotechnology, National Agricultural and Food Research and Technology Institute (INIA), Spanish National Research Council (CSIC), Madrid, Spain

**Keywords:** probiotics, *Bacillus subtilis*, infectious pancreatic necrosis virus (IPNV), VP2, vaccines, rainbow trout

## Abstract

*Bacillus subtilis* has been documented in the past years as an effective probiotic for different aquacultured species, with recognized beneficial effects on water quality, fish growth and immune status. Furthermore, its potential as a vaccine adjuvant has also been explored in different species. In the current work, we have used *B. subtilis* spores as delivery vehicles for the presentation of the VP2 protein from infectious pancreatic necrosis virus (IPNV). For this, the VP2 gene was amplified and translationally fused to the crust protein CotY. The successful expression of VP2 on the spores was confirmed by Western blot. We then compared the immunostimulatory potential of this VP2-expressing strain (CRS208) to that of the original *B. subtilis* strain (168) on rainbow trout (*Oncorhynchus mykiss*) leukocytes obtained from spleen, head kidney and the peritoneal cavity. Our results demonstrated that both strains significantly increased the percentage of IgM^+^ B cells and the number of IgM-secreting cells in all leukocyte cultures. Both strains also induced the transcription of a wide range of immune genes in these cultures, with small differences between them. Importantly, specific anti-IPNV antibodies were detected in fish intraperitoneally or orally vaccinated with the CRS208 strain. Altogether, our results demonstrate *B. subtilis* spores expressing foreign viral proteins retain their immunomodulatory potential while inducing a significant antibody response, thus constituting a promising vaccination strategy.

## Introduction

*Bacillus* ssp. have been widely tested as probiotics in fish not only searching to increase survival upon pathogen encounter but also aiming to reduce animal stress, promote animal growth, and improve feed conversion and water quality (1). Particularly, *Bacillus subtilis* has been widely tested as a probiotic in aquaculture in the last years due to its advantageous characteristics. This bacterium is ubiquitous thanks to its ability to secrete enzymes, metabolites and antibiotics that benefit its nutrient uptake, while having a negative impact on other competitive microbes (2). Furthermore, most strains of *B. subtilis* are non-pathogenic, non-hemolytic and do not present antibiotic resistance (3–5). Although *B. subtilis* is a spore-producer and is considered a facultative anaerobe, it can expand in the digestive tract, where it secretes antimicrobial compounds, enzymes and polymers that interfere with pathogenic bacteria and condition its probiotic effects (4, 6).

Consequently, experiments performed in different fish species have demonstrated that the oral administration of *B. subtilis* decreases the mortality provoked by exposure to different bacterial pathogens (5, 7–9). This increased protection is in great part mediated by the immunostimulatory effects exerted by the probiotic. Thus, fish fed for several weeks with *B. subtilis*-supplemented diets showed increased IgM titers (9), lysozyme activity (5, 10), bactericidal activity (3, 10) or complement activity (10) in serum. Recent studies have also demonstrated that the phagocytic activity of head kidney leukocytes is significantly enhanced in response to *B. subtilis*-supplemented diets (10). Finally, in rainbow trout, the oral administration of *B. subtilis* also up-regulated the levels of transcription of interleukin-1β (IL-1β), tumor necrosis factor α (TNFα) and transforming growth factor β (TGFβ) in the spleen and head kidney (10). In our laboratory, we have very recently established that *B. subtilis* induces the transcription of a wide range of immune genes in rainbow trout (*Oncorhynchus mykiss*) intestinal epithelial cells and intestine explants (in press). Finally, our studies have also demonstrated the capacity of this probiotic to promote the survival of B cells and increase the amount of IgM-secreting cells in rainbow trout splenic leukocytes (in press). In addition to its immunostimulating capacities, recent studies have successfully used *B. subtilis* spores as an immunization vehicle (11–15). This approach provides a robust method for the delivery of antigens, beneficiating from the immunostimulatory properties of the vehicle itself.

Infectious pancreatic necrosis virus (IPNV) is a non-enveloped and segmented doubled-stranded RNA virus that belongs to the *Aquabirnavirus* genus within the *Birnaviridae* family (16). Its genome is divided in two segments, A and B. Segment A contains two partially overlapping open reading frames (ORFs), the larger encoding the structural proteins VP2 and VP3 and a protease (VP4). A smaller ORF codes for the nonstructural protein VP5. Segment B codes for the RNA-dependent RNA polymerase (17). All neutralizing antibodies against IPNV are specific to VP2 (18). The virus causes IPN, a disease that generates high mortality rates along with tremendous economic losses in salmonid aquaculture, especially affecting fry and juvenile fish (19). Although several vaccination strategies have been developed in the past years to prevent IPN, the virus is still responsible for important economic losses in rainbow trout and salmon farms worldwide (20).

Thus, in the current study, we have explored the possibility of using *B. subtilis* spores as a vehicle to immunize rainbow trout against IPNV. We have not only confirmed that the recombinant VP2-expressing *B. subtilis* strain was capable of inducing a specific antibody response but we have also undertaken a series of experiments *in vitro* using splenic, head kidney and peritoneal leukocytes to confirm that the immunostimulatory capacity of *B. subtilis* spores was retained despite the VP2 expression. Our findings demonstrate that *B. subtilis* spores expressing a viral antigen maintain their immunostimulatory properties while eliciting an adequate specific antibody response, pointing to this as a promising vaccination strategy for fish that could be applied either intraperitoneally or orally given the capacity of *B. subtilis* spores to tolerate a low pH and high bile concentration (21, 22).

## Materials and Methods

### Production of VP2-Producing *B. subtilis* Spores

**Figure 1:**
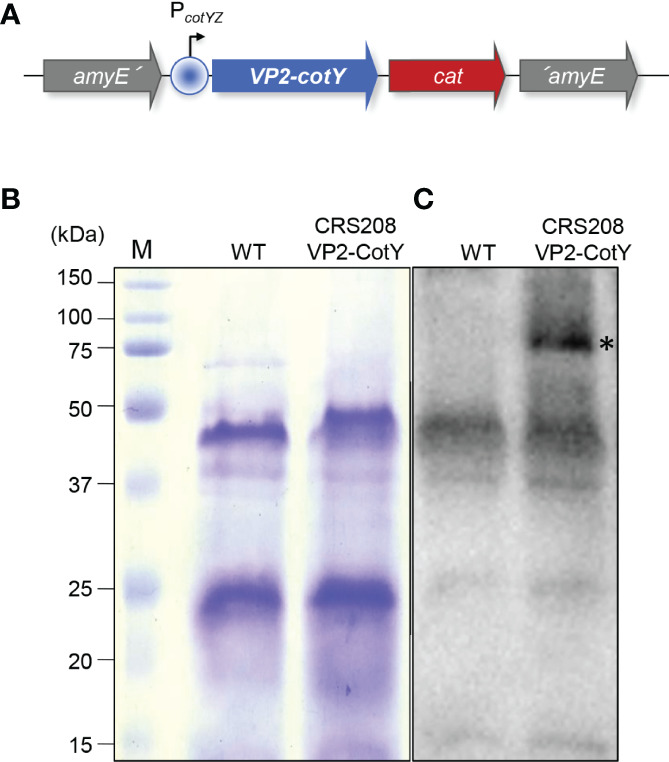
**(A)** Schematic representation of the CRS208 genotype. The VP2-encoding gene was translationally fused to the spore protein-encoding gene *cotY*, under the control of the *cotYZ* promoter, and integrated at the non-essential *amyE* locus of *B. subtilis* 168, creating the CRS208 strain. **(B)** Spore proteins from the parental strain *B. subtilis* 168 (WT) and its congenic derivative CRS208 were separated in a 15% acrylamide gel with 6 M of urea, revealing a similar pattern. **(C)** Western blot analysis using an anti-VP2 primary antibody (1:1000) detected a band of approximately 74 kDa (highlighted by an asterisk) equivalent to the sum of the MW of CotY (18 kDa) and VP2 (56 kDa), that was absent in the protein extracts from WT spores.

A 1475 bp DNA fragment containing the coding sequence of the VP2 of the IPNV NVi-015 strain was PCR amplified from plasmid pMCV1.4 VP2015 (23), using oligonucleotide primers cotY-VP2-F and cotY-VP2-R (**Table S1**) and the Phusion™ High-Fidelity DNA Polymerase (Thermo Scientific). The DNA fragment was sequentially digested with NgoMIV and SpeI (Thermo Scientific) and inserted into the p1CSV-CotY-C plasmid (24) previously cleaved with SpeI and AgeI, yielding pGG4 (genotype: *bla amyE3´ PcotYZ-vp2-cotY cat amyE5´*). Transformation of *B. subtilis* 168 competent cells with ScaI-linearized pGG4 originated the chloramphenicol resistant strain CRS208 (genotype: amyE: PcotYZ-vp2-cotY, CmR). The correct chromosomal integration was checked by PCR using primers amyE-F and amyE-R (**Table S1**; **Figure 1A**).

Spores of *B. subtilis* 168 and its congenic derivative CRS208 were obtained by nutrient exhaustion in DSM (Difco Sporulation Medium), purified and quantified as described before (24, 25). To verify the correct expression of the VP2, spore suspensions were mixed with 2x loading buffer (10% glycerol, 10% 2-mercaptoethanol, 100 mM dithiothreitol (DTT), 4% SDS, 0.05% bromophenol blue and 0.125 M Tris), boiled for 8 min and spore proteins were resolved by SDS-PAGE on a 15% acrylamide gel containing 6 M of urea. After electrotransfer to a 0.2 µm nitrocellulose membrane, spore proteins were detected by Western blot analysis with a rabbit anti-VP2 polyclonal antibody (1:1000) followed by a 1:10000 goat anti-rabbit IgG (H+L) horseradish peroxidase (HRP) conjugated antibody (Thermo Scientific, catalogue number 31466). Visualization was carried out in a ChemiDoc XRS Gel Imaging System (Bio-Rad) and analyzed with the Image Lab Software (Bio-Rad).

### Experimental Fish

Rainbow trout (*Oncorhynchus mykiss*) of approximately 100 g (used for leukocyte isolation) and 30 g (used for the intraperitoneal injection *in vivo* assay) were obtained from Cifuentes fish farm (Cifuentes, Guadalajara, Spain). Rainbow trout of approximately 10 g (used for the oral administration *in vivo* assay) were obtained from Felechosa fish farm (Felechosa, Asturias, Spain). In all cases, fish were maintained in an aerated recirculating system at 15°C with 12:12 h light/dark photoperiod and were fed twice a day with a commercial diet (Skretting Spain, S.A). Fish were acclimatized to laboratory conditions for at least two weeks prior to any experimental procedure, and during this period, no clinical signs were ever observed.

All the experiments described comply with the Guidelines of the European Union Council (2010/63/EU) for the use of laboratory animals and have been approved by the INIA Ethics Committee (Code PROEX 153/17).

### Leukocyte Isolation

Rainbow trout were sacrificed with benzocaine (Sigma) overdose administered by immersion (50 mg/l) following the recommendations of Zahl et al. (26). Total leukocyte populations were then isolated from spleen, head kidney and the peritoneal cavity. For this, the abdomen was disinfected with 70% ethanol and the peritoneal cavity was injected with 2 ml of Leibovitz medium (L-15, Life Technologies) supplemented with 100 I.U./ml penicillin, 100 µg/ml streptomycin (P/S, Life Technologies), 10 U/ml heparin (Life Technologies) and 2% fetal calf serum (FCS, Life Technologies). After gently massaging the abdominal surface, the medium containing the peritoneal cells was harvested from the peritoneum inserting a pipette tip through a peritoneal incision. The peritoneal cavity was then opened and 2 ml of medium added to collect any remaining cells. At this point, the spleen and the head kidney were also collected. Single cell suspensions were also obtained from these tissues after disaggregation using 100 µm nylon mesh strainers and the same media. The suspension containing peritoneal cells was also passed through a 100 µm nylon mesh strainer. All cell suspensions were then placed onto 30/51% discontinuous Percoll (GE Healthcare) density gradients and centrifuged at 500 x g for 30 min at 4°C, without brake. The interface cells were collected and washed in L-15 containing P/S and 2% FCS. The viable cell concentration was determined by Trypan blue (Sigma-Aldrich) exclusion on a Neubauer chamber. Cells were resuspended in L-15 supplemented with P/S and 5% FCS and were initially adjusted to 1x10^6^ cells/ml.

### Flow Cytometry Analysis

To evaluate the effect of the bacterial strains on the B cell subpopulations, leukocytes from peritoneum, spleen and head kidney were dispensed into 96-well plates (4x10^5^ cells per well) and were incubated for 48 h at 20°C with the *B. subtilis* strains 168 and CRS208, at a final concentration of 1x10^7^ cfu/ml. Controls without bacteria were also included.

After the stimulation, the percentage of IgM^+^ B cells in the leukocyte cultures was estimated through flow cytometry. For this, leukocytes were incubated with a specific monoclonal antibody against rainbow trout IgM [1.14 mAb mouse IgG1 coupled to R-phycoerythrin (R-PE), 1 µg/ml] in staining buffer (phenol red-free L-15 medium supplemented with 2% FCS) for 1 h at 4°C. The anti-trout IgM used in this study has been previously characterized (27) and was fluorescently labeled using the R-PE Lightning-Link labeling kit (Innova Biosciences) following manufacturer’s instructions. After the incubation with anti-IgM, leukocytes were washed two times with staining buffer and counterstained with 0.2 µg/ml 4’,6-diamidino-2-phenylindole (Sigma), to identify dead cells and discard them from the analysis. An isotype control was tested in parallel to discard unspecific binding of the anti-IgM (R-PE -mouse IgG1, Biolegend). Samples were analyzed on a FACS Celesta™ flow cytometer (BD Biosciences) equipped with BD FACSDiva software. Flow cytometry analysis was performed with FlowJo^®^ v.10 (FlowJo LLC, Tree Star).

### ELISpot

ELISpot was used to quantify the number of total IgM-secreting B cells in peritoneal, splenic and head kidney leukocytes. For this, leukocytes dispensed in 96-well plates (1x10^4^ cells/well for peritoneal and 5x10^4^ cells/well for spleen and head kidney leukocytes) were incubated with 1x10^7^ cfu/ml of the two *B. subtilis* strains, 168 and CRS208, for 48 h at 20°C. During this time, ELISpot plates containing Immobilon-P membranes (Millipore) were activated with 70% ethanol and coated with 2 µg/ml of an anti-trout IgM mAb (clone 1.14) for 48 h at 4°C in agitation. Non-specific binding sites were blocked by incubation with 2% BSA (bovine serum albumin) in PBS (phosphate buffer saline) for 2 h at room temperature (RT). After the incubation, cells were transferred to ELISpot plates coated with an anti-IgM mAb and incubated for a further 24 h at 20°C. Cells were then washed 5 times with PBS and plates blocked with 2% BSA in PBS for 1 h at RT. After blocking, biotinylated anti-trout IgM mAb (clone 1.14) was added to the plates (1 μg/ml) that were subsequently incubated for 1 h at RT. Following additional washing steps (5 times in PBS), the plates were developed using streptavidin-HRP (Thermo Fisher Scientific) (100 ng/ml) for 1 h at RT, washed again with PBS and incubated with 3-amino 9-ethylcarbazole (Sigma-Aldrich) for 30 min at RT in the dark. The substrate reaction was stopped by washing the plates with tap water. Once the membranes were dried, the number of spots in each well was determined using an AID iSpot Reader System (Autoimmun Diagnostika GMBH).

### Gene Expression Analysis

Leukocytes dispensed in 24-well plates (2x10^6^ cells/well in the case of spleen and head kidney and 1x10^5^ cells/well for peritoneal leukocytes) were incubated with 1x10^7^ cfu/ml of the two *B. subtilis* strains, 168 and CRS208, for 24 h at 20°C.

Total RNA was extracted from the spleen and head kidney leukocytes using TRI Reagent solution (Invitrogen) according to the manufacturer’s instructions and then quantified using a NanoDrop 1000 Spectrophotometer (Thermo Fisher Scientific). RNA was treated with DNase during the process to remove genomic DNA that might interfere with the PCR reactions. cDNA was obtained from 1 µg of total RNA using RevertAid Reverse Transcriptase (Thermo Fisher Scientific), primed with oligo(dT)_23_VN, following manufacturer’s instructions. To evaluate gene transcription levels, real-time PCR was performed in a LightCycler96 System instrument (Roche) using FastStart Essential DNA Green Master reagents (Roche) and specific primers (**Table S2**). Each sample was subjected to the following conditions: 10 min at 95°C, followed by 40 amplification cycles (10 s at 95°C, 10 s at 60°C and 10 s at 72°C). A dissociation curve was obtained by reading fluorescence every degree between 60°C and 95°C to ensure only a single product had been amplified.

In the case of peritoneal leukocytes, total RNA was isolated using the Power SYBR Green Cells-to-Ct Kit (Invitrogen) following manufacturer´s instructions. RNA was also treated with DNase during the process. Reverse transcription was also performed using the Power SYBR Green Cells-to-Ct Kit (Invitrogen) following manufacturer’s instructions. To evaluate the levels of transcription of the different genes, real time PCR was performed with a LightCycler 96 System instrument using SYBR Green PCR core Reagents (Applied Biosystems) and specific primers (**Table S2**). Each sample was measured in duplicate under the following conditions: 10 min at 95°C, followed by 40 amplification cycles (15 s at 95°C and 1 min at 60°C). A melting curve for each primer set was obtained by reading fluorescence every degree between 60°C and 95°C to ensure only a single product had been amplified.

In all cases, the relative expression levels of the genes were normalized to the expression of elongation factor 1α (EF-1α), as a housekeeping gene control following the MIQE guidelines (28). This housekeeping gene was selected after verifying that no statistical differences were detected among EF-1α Ct values, obtained from different samples. Expression levels were calculated using the 2^-ΔCt^ method, where ΔCt is determined by subtracting the EF-1α value from the target cycle threshold. Negative controls with no template and *minus*-reverse transcriptase controls were included in all cases.

### *In Vivo* Assays

Rainbow trout of approximately 30 g were intraperitoneally injected with 100 µl of saline solution (0.9% NaCl) containing 2x10^8^ spores of the CRS208 *B. subtilis* strain expressing the IPNV VP2 protein. A mock immunized group (control) received 100 µl of saline solution. In another experiment, 100 µl containing 2x10^9^ spores of the CRS208 *B. subtilis* strain were orally administered by force-feeding to rainbow trout of approximately 10 g average weight. The same volume of saline solution was orally administered to the control group.

In both cases, at day 30 post-immunization, 9 fish from each group were sacrificed by benzocaine overdose and blood extracted from the caudal vein. Blood removed from the caudal vein of experimental fish was let to clot at 4°C overnight. Serum extraction was then performed by centrifugation at 4000 x g for 10 min at 4°C and the supernatant were centrifuged again at 10000 x g for 10 min at 4°C. Serum was stored at -80°C until use.

### ELISA

The presence of IPNV-specific trout IgM in serum of immunized fish was estimated by ELISA following a protocol previously described by Munang’andu *et al.* (29). For this, plates were coated with 100 μl of polyclonal anti-IPNV antibody diluted at 1:5000 in diluent buffer (0.05 M carbonate buffer pH 9.7) and incubated overnight at 4°C. Plates were washed 3 times in PBS-0.05% Tween-20 (PBT) and blocked with 5% fat free dry milk-PBS for 2 h at RT. Viral antigen of the T_T217_A_221_ IPNV strain was added to each well after incubation at RT for 2 h. Diluted sera in 1% dry milk 5% fat free dry milk-PBS were added to each well. Blank and positive controls were also included. After incubation at 4°C overnight, each well was incubated with 1 μg/ml biotinyilated anti-trout IgM mAb (clone 1.14) diluted in 5% fat free dry milk-PBS for 1 h at RT. The plates were washed again three times in PBT and 100 ng/ml of HRP-streptavidin (Thermo Fisher Scientific) added to each well in 100 μl 5% fat free dry milk-PBS. After incubation at RT for 1 h, 100 μl of o-phenylenediamine dihydrochloride substrate reagent (Sigma-Aldrich) were added to each well. The reaction was stopped after 15 min by adding 50 μl of 2.5 M H_2_SO_4_. Absorbances were recorded at 490 nm using a FLUOstar Omega (BMG Labtech) plate reader. Internal positive and negative control samples were included in all assays.

### Statistical Analysis

Data handling, statistical analyses and graphic representation were performed using GraphPad Prism version 8 (GraphPad Software). The assumption of normality was checked using Shapiro-Wilk test. Non-normal data sets were transformed using a ln(Y). When two groups were compared, statistical analysis were performed using a paired two-tailed Student’s *t*-test or an unpaired Student’s t test with Welch’s correction. Differences in transcriptional analysis were analyzed by ANOVA and *post hoc* comparison was performed using the Tukey´s multiple comparison test. The differences between the mean values were considered significant on different degrees, where * means *p* ≤ 0.05, ** means *p* ≤ 0.01 and *** means *p* ≤ 0.001.

## Results

### Production of Recombinant *B. subtilis* Expressing the IPNV VP2 Protein

A recombinant *B. subtilis* strain (CRS208) expressing the VP2 of IPNV was obtained from the *B. subtilis* 168 wild type strain. Spore proteins from both strains were first separated on an acrylamide gel, revealing a similar protein pattern (**Figure 1B**). The correct expression of the IPNV VP2 by the CRS208 strain was then verified by Western blot. A band of approximately 74 kDa equivalent to the sum of the MW of CotY (18 kDa) and VP2 (56 kDa) was detected by VP2 specific antibody on the extracts of the recombinant CRS208 strain (**Figure 1C**).

### *B. subtilis* 168 and CRS208 Strains Promote the Survival of IgM^+^ B Cells

**Figure 2:**
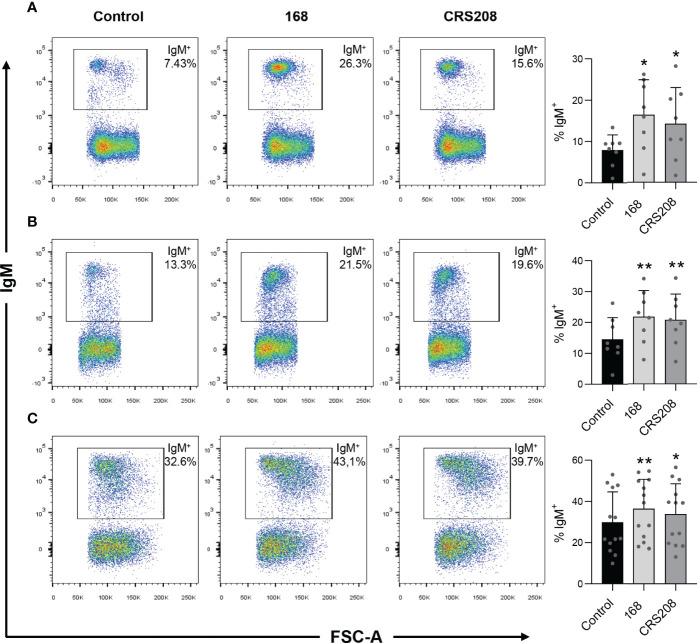
Effect of *B. subtilis* 168 and CRS208 strains on the percentage of IgM^+^ B cells. The percentage of IgM^+^ B cells was measured *via* flow cytometry using a specific anti-IgM in spleen **(A)**, kidney **(B)** and peritoneal **(C)** leukocyte cultures treated with 1x10^7^ cfu/ml *B. subtilis* 168 or CRS208 strains for 48 h. Controls not treated with bacteria were also included. Representative dot plots are included along with graphs showing the percentage of live IgM^+^ B cells in the lymphocyte gate (mean + SD; n=8). Statistical analyses were performed using a paired two-tailed Student’s *t*-test, after checking the normality using the Shapiro-Wilk test. Asterisks denote significant differences in stimulated cultures compared to control cultures, where *p ≤ 0.05 and **p ≤ 0.01.

To confirm that the immunogenic potential of the recombinant strain remained similar to that of the wild type *B. subtilis* strain, we first evaluated the effects of the transformed *B. subtilis* strain expressing the IPNV VP2 protein (CRS2018) and the original non-transformed *B. subtilis* strain (168) on the percentage of naive B cells. For this, spleen, head kidney and peritoneal leukocytes were incubated in the presence or absence of the *B. subtilis* 168 or CRS208 strains for 48 h in standard culture conditions. The flow cytometric analysis of these populations was conducted following the gating strategy described in **Figure S1**. Our results show a significant increase in the percentage of IgM^+^ B cells in leukocyte cultures treated with both the *B. subtilis* 168 strain and the CRS208 strain in comparison to control leukocytes (**Figure 2A**). This increase was clear in the case of spleen (**Figure 2A**), head kidney (**Figure 2B**) and peritoneal (**Figure 2C**) leukocytes and was also evident when the absolute numbers of IgM^+^ cells were calculated among a fixed number of live gated cells (**Figure S2**). No significant differences were found between the effects promoted by the two strains in either case (**Figure 2**; **Figure S1**), demonstrating that the expression of a foreign antigen on *B. subtilis* does not have a negative effect on the viability of B cells.

### *B. subtilis* Strains Induce IgM Secretion in Leukocyte Cultures

**Figure 3:**
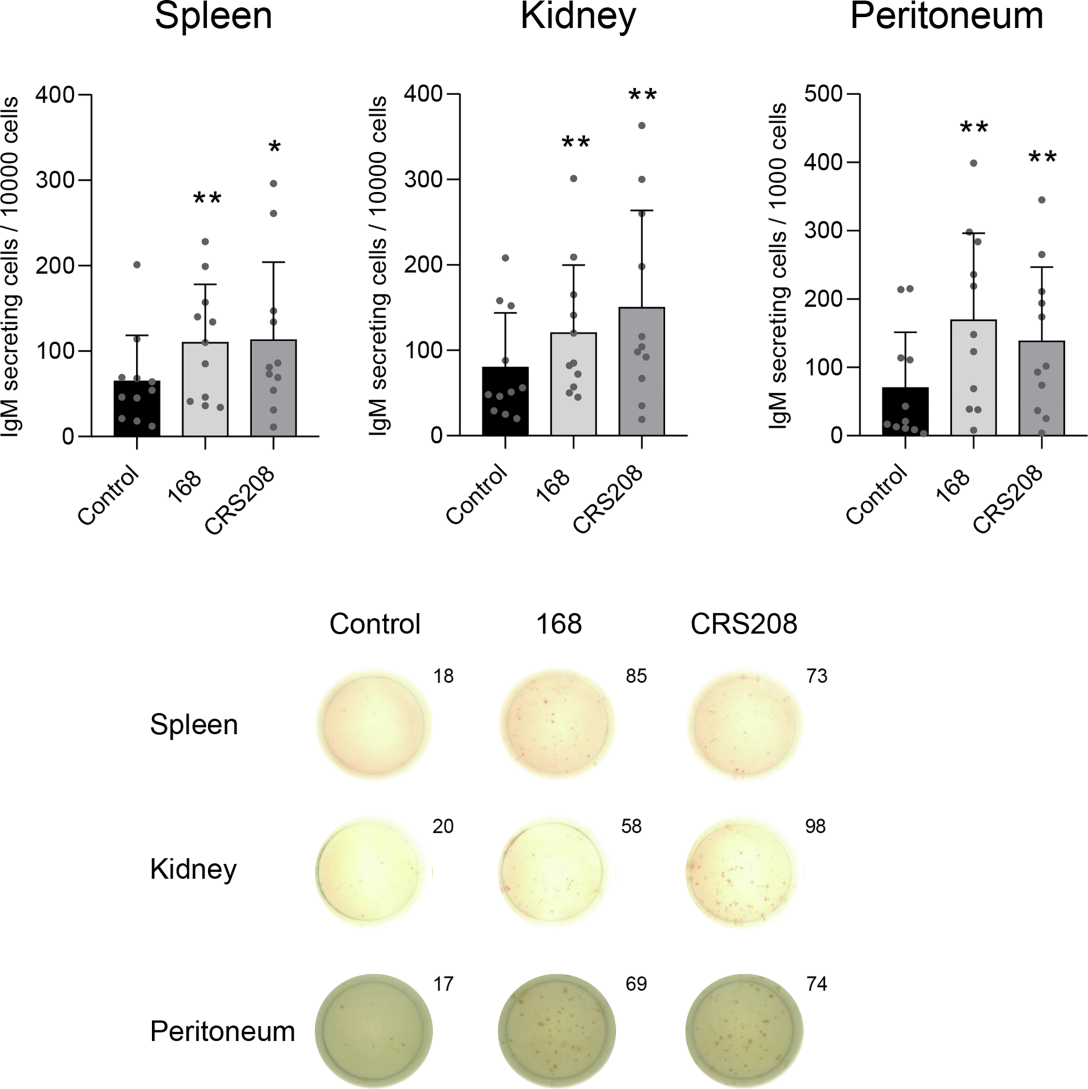
Effect of *B. subtilis* 168 and CRS208 strains on the number of IgM-secreting cells. ELISpot analysis was used to estimate the number of IgM-secreting cells in spleen, kidney and peritoneal leukocytes incubated with 1x10^7^ cfu/ml *B. subtilis* 168 or CRS208 strains. Controls not treated with bacteria were also included. Cells were cultured for 48 h and then plated in ELISpot plates, previously coated with anti-IgM for a further 24 h. Quantification of spot forming cells are shown (mean + SD; n=12) along with representative wells from one individual (below). Statistical analyses were performed using paired a two-tailed Student’s *t*-test of ln(Y) transformed data. The normal distribution was confirmed using the Shapiro-Wilk test. Asterisks indicate significant differences in stimulated cultures compared to control cultures, where *p ≤ 0.05 and **p ≤ 0.01.

Next, we assessed whether the *B. subtilis* strains could have an effect on the number of cells secreting IgM after stimulating spleen, head kidney and peritoneal leukocytes with the different strains for 48 h. The number of IgM-secreting cells was then determined by ELISpot. When leukocytes were treated with either one of the *B. subtilis* strains, a significant increase in the number of IgM-secreting cells was observed in comparison to that obtained in control cultures (**Figure 3**). Again, this increase occurred in in leukocyte cultures obtained from spleen, head kidney and peritoneum (**Figure 3**). No significant differences were found between the effects promoted by the two strains.

### Transcriptional Changes Induced by *B. subtilis* Strains in Spleen, Head Kidney and Peritoneal Leukocytes

**Figure 4:**
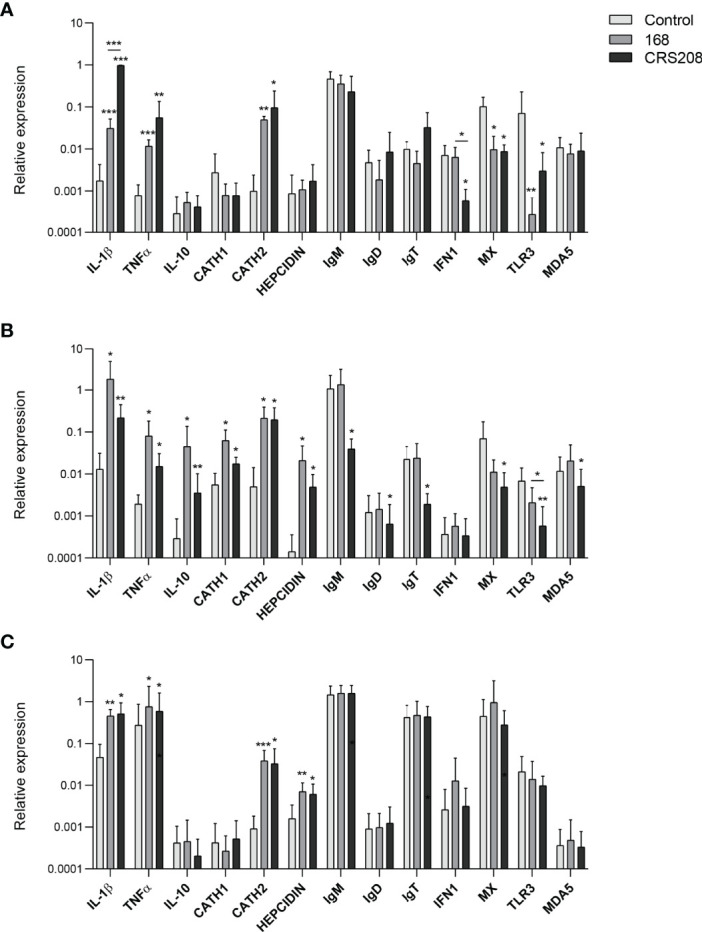
Transcriptional response of leukocytes to *B. subtilis* strains 168 and CRS208. Spleen **(A)**, head kidney **(B)** and peritoneal **(C)** leukocytes were incubated with 1x10^7^ cfu/ml of *B. subtilis* 168 or CRS208 during 24 h at 20°C. Controls not treated with bacteria were also included. RNA was extracted and the levels of transcription of different genes analyzed by real time PCR. Data are shown as the mean relative gene expression normalized to the transcription of the housekeeping gene EF-1α + SD (n=8). Statistical analyses were performed by ANOVA and a *post hoc* comparison by Tukey´s multiple comparison test. The normal distribution was confirmed using the Shapiro-Wilk test, and non-normal data were transformed using a ln(Y). Asterisks denote significant differences in stimulated cultures compared to control cultures, and underlined asterisks show significant differences between the two bacterial strains, where *p ≤ 0.05, **p ≤ 0.01 and ***p ≤ 0.001.

To further investigate the immunostimulatory potential of the two *B. subtilis* strains, we evaluated their effects on the transcriptional levels of several immune genes in leukocyte cultures. Therefore, after leukocytes from spleen, kidney and peritoneum were stimulated with the different strains for 24 h at 20°C, we assessed the levels of transcription of a wide range of immune genes, including pro-inflammatory genes (IL-1β and TNF-α), the anti-inflammatory cytokine IL-10, antimicrobial peptides (cathelicidin 1 and 2 and hepcidin), fish Igs (IgM, IgD and IgT) and genes involved in the antiviral response such as interferon 1 (IFN1), Mx, toll-like receptor 3 (TLR3) and melanoma differentiation-associated protein 5 (MDA5). Interestingly, the response to the bacteria slightly differed among leukocytes from different sources. Thus, spleen leukocytes treated with either of the two strains showed significantly higher levels of transcription of IL-1β, TNF-α and cathelicidin 2 compared with the untreated cells. On the contrary, splenic leukocytes treated with either the *B. subtilis* 168 or the CRS208 strain, significantly down-regulated the transcription of Mx and TLR3, and also that of IFN1 in the case of the CRS208-treated splenocytes (**Figure 4A**).

More genes were transcriptionally activated by the *B. subtilis* strains in head kidney leukocytes (**Figure 4B**). Thus, both strains significantly up-regulation the transcription of the IL-1β, TNF-α, IL-10, cathelicidin 1, cathelicidin 2 and hepcidin compared to control cells (**Figure 4B**). Surprisingly, despite the positive effects provoked by the bacteria in B cell survival and IgM secretion, at a transcriptional level, the CRS208 strain significantly decreased the mRNA levels of all the Igs in head kidney leukocytes (**Figure 4B**). Finally, the levels of transcription of Mx, TLR3 and MDA5 were also significantly down-regulated in leukocytes exposed to the CRS208 strain (**Figure 4B**).

Less transcriptional changes were observed in response to the bacteria in the case of peritoneal leukocytes (**Figure 4C**). Both strains had the capacity to significantly increase the transcription of IL-1β, TNF-α, cathelicidin 2 and hepcidin in comparison to control cells (**Figure 4C**).

Therefore, the ability of the two bacterial strains to modulate the transcription of different genes involved in immune response has been clearly demonstrated and only a few significant differences were observed when comparing the effects of the two strains. Thus, the CRS208 strain increased IL-1β transcription at levels significantly higher than those induced by the 168 strain in the spleen. In contrast, the CRS208 strain significantly reduced IFN1 transcription in the spleen and TLR3 in the head kidney, when compared to the levels observed in control cultures and in cultures treated with the 168 strain.

### The CRS208 *B. subtilis* Strain Induces IPNV-Specific Antibodies

**Figure 5:**
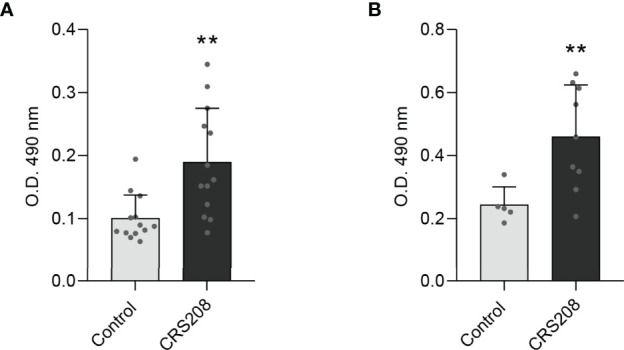
Detection of IPNV-specific IgM in fish serum after intraperitoneal injection **(A)** or oral administration **(B)** of the *B. subtilis* CRS208 strain. The concentration of IPNV-specific IgMs was evaluated as described in Materials and Methods in serum samples obtained from immunized or mock-immunized fish at day 30 post-immunization. Results are shown as mean values of absorbance at 490 nm (n = 9). Statistical analysis of the data were performed using an unpaired two-tailed Student’s *t*-test with Welch’s correction. The normal distribution was confirmed using the Shapiro-Wilk test. Asterisks denote significantly different values among groups as indicated (**p ≤ 0.01).

Finally, we verified that the transformed *B. subtilis* strain expressing the IPNV VP2 protein was capable of inducing IPNV-specific IgM titers when intraperitoneally injected in rainbow trout (**Figure 5A**) or after a single oral administration (**Figure 5B**). No specific antibodies were detected in fish injected with saline solution (**Figures 5A, B**) nor in fish injected with the 168 non-transformed *B. subtilis* strain. The mean values of absorbance at 490 nm obtained in fish intraperitoneally injected with the non-transformed *B. subtilis* strain were even lower than those obtained in fish injected with the saline solution (mean value of 0.082; n=9).

## Discussion

In aquaculture, mucosal immunization has several advantages over systemic vaccination. First of all, as the higher capacity of mucosal immunization to trigger mucosal immunity than that of systemic immunization has been broadly demonstrated (30–33), an effective response established at these mucosal sites through this type of vaccination would block pathogens at these entry sites before they colonize the host (20, 30, 34, 35). On the other hand, oral delivery or immersion of vaccines in fish farms imply less economic costs and reduced fish stress while it facilitates vaccination at early developmental stages when fish are more susceptible to infections (32, 36, 37). However, the development of oral vaccines has been proven a challenging task. First, oral vaccines need to avoid degradation in the stomach to reach the distal responsive segments of the intestinal tract unaltered, where they can be taken up by antigen presenting cells. Additionally, they need to breach the very restricted tolerogenic immune mechanisms that tightly regulate the gut responses, taking into account that the gut is continuously exposed to microbiota or food antigens against which immune responses should be avoided (34).

In this context, probiotics have great potential to be engineered as oral vaccine delivery strategies. First, they have the capacity to colonize the mucosal surface of the intestinal tract (38), reaching the more distal segments. In contrast to what happens when potentially pathogenic bacteria are used as vaccine vehicles, probiotics do this without provoking a strong immune reaction against them (13). Nevertheless, the immunomodulatory capacity that some of these probiotics have on the host, widely documented for many probiotic species (1, 39), can cooperate during the onset of adaptive immune responses, thereby acting somehow as an intrinsic adjuvant (40, 41).

Among the different bacterial species used as probiotics, spore-formers show critical advantages for application in aquafeeds (42), as the bacterial spores can be easily produced on a large scale and be dehydrated, allowing long-term storage without losing characteristics, facilitating feed incorporation. Moreover, the capacity to produce spores is not only important from the production point of view, but also because spores allow bacteria to survive the harsh conditions of the stomach, becoming successfully established in the intestine (43). In this context, *B. subtilis* is an ideal candidate to use as a vaccine vehicle, given the robustness of the spores it produces, its easy gene operability, its safety and its intrinsic immunomodulatory capacity (44). Thus, recently, several studies have addressed the use of *B. subtilis* spores as an immunization vehicle, through the expression of pathogenic antigens on their surface. Hence, *B. subtilis* spores expressing the VP4 of a grass carp reovirus, when administered orally, were able to increase the survival of grass carp *(Ctenopharyngodon idella*) to a subsequent viral infection (13). In another study, the effectiveness of *B. subtilis* spores expressing the grass carp reovirus VP4 protein and that of spores expressing the NS38 protein was compared. Although similar protection rates were obtained with the two recombinant *B. subtilis* strains, the mechanisms through which they exerted these effects were very different. Thus, while the VP4-expressing strain mainly elicited specific systemic and mucosal antibody responses, the NS38-expressing strain mainly induced an inflammatory response, while promoting cellular immunity (14). *B. subtilis* spores have also been recently shown to reduce the infection of fish with *Clonorchis sinensis*, an important fish-borne zoonotic parasite. For example, the administration of *B. subtilis* spores expressing the *C. sinensis* enolase stimulated both innate and adaptive responses, as well as mucosal and systemic responses in grass carp (11). Similarly, *B. subtilis* spores expressing the *C. sinensis* cysteine protease increased IgM levels in serum and intestinal mucus from treated grass (12). Finally, the administration of *B. subtilis* spores expressing the parasite´s paramyosin protein, also induced a specific immune response and significantly reduced the amount of metacercaria in the fish flesh (15).

In the current study, we have produced *B. subtilis* spores expressing the VP2 protein of IPNV. Although there are some vaccines against IPNV available in the market, these are all delivered by injection and their effectiveness in field conditions is still limited as the virus continues to provoke important economic losses every year in salmonid farming (20, 45, 46). Different oral vaccination strategies have been recently attempted against IPNV. These include DNA vaccines coding for the IPNV VP2 capsid protein, encapsulated in alginate (47–51) or liposomes (52). The use of bacteria expressing IPNV was also attempted using *Lactobacillus casei* and tested in rainbow trout orally immunized, reducing viral loads after IPNV challenge (14, 53–56). However, the possibility of obtaining an IPNV vaccine based on a spore-forming probiotic had never been addressed.

Initially, we tested the immunostimulatory potential of this VP2-expressing strain (CRS208) to that of the original *B. subtilis* strain (168) on rainbow trout leukocytes obtained from spleen, head kidney and the peritoneal cavity, as we believe that the immunomodulatory capacity that *B. subtilis* exerts on the host is important to elicit a robust immune response. Concerning the effects on B cells, our results demonstrated that the CRS208 strain significantly increased the survival of IgM^+^ B cells and the number of IgM-secreting cells in all leukocyte cultures at similar levels than those induced by the 168 strain. Furthermore, we determined the transcriptional response of leukocytes obtained from different sources to the bacteria. Interestingly, in general, the transcriptional changes induced by either of the *B. subtilis* strains tested were more numerous in head kidney leukocyte cultures than those observed in leukocyte cultures from the peritoneum or the spleen. In leukocytes from all sources, *B. subtilis* (either CRS208 or 168) consistently increased the levels of transcription of IL-1β, TNF-α and cathelicidin 2, whereas other pro-inflammatory cytokines and antimicrobial peptides were also induced by the bacterial strains in specific leukocyte cultures. Although the transcriptional profile elicited by both *B. subtilis* strains was similar, there were some differences between CRS208 and its wild type 168. Thus, the CRS208 strain induced IL-1β transcription at levels significantly higher than those induced by the 168 strain in the spleen. In contrast, the CRS208 strain significantly reduced IFN1 transcription in the spleen and TLR3 in the head kidney, in comparison to the levels observed in control cultures and in cultures treated with the 168 strain. The different response of these two genes involved in the viral response to the bacterial strain CRS208 expressing the IPNV VP2 protein is an interesting result, that needs to be further explored, but could be related to the fact that different IPNV proteins, including VP2, have the capacity to inhibit IFN production (57). This capacity of IPNV VP2 to interfere with the host immune response could also be at least in part responsible for the significant reductions of the levels of transcription of many immune genes that were provoked by the CRS208 strain in head kidney, but of course this is also something that needs to be confirmed in future studies.

Having established that the VP2-expressing *B. subtilis* strain (CRS208) retains the immunomodulatory potential of the original *B. subtilis* strain (168), we then tested the capacity of this strain to induce IPNV-specific antibodies. This capacity was confirmed first through an intraperitoneal injection, and then after a single oral administration. Although the efficacy of this prototype IPNV vaccine should be further confirmed determining the protection conferred after a challenge, the fact that a significant specific humoral antibody response is mounted is a good indication that of its efficacy as previously reported (35, 56).

In summary, the immunomodulatory properties and the immunogenicity of recombinant *B. subtilis* spores expressing the VP2 of IPNV have been clearly revealed. This new recombinant bacterial strain has been proven to properly express the antigen, being capable of inducing a significant antibody response after a single administration. This response was elicited even upon an oral administration demonstrating that *B. subtilis* spores are gastro-resistant vaccine vehicles, that reach undisturbed the most distal segments of the gut where the antigen can be recognized and immune responses triggered. These results further support the great potential of *B. subtilis* spores as vaccine vehicles for the design of oral vaccines for use in aquaculture.

## Data Availability Statement

The raw data supporting the conclusions of this article will be made available by the authors, without undue reservation.

## Ethics Statement

The animal study was reviewed and approved by the INIA Ethics Committee (Code PROEX 153/17).

## Author Contributions

FD: Data curation, formal analysis, investigation, software, writing - review and editing. NN-O: Data curation, formal analysis, investigation, writing – review and editing. GG: Resources, investigation. CS: Resources, writing – review and editing. EG-C: Conceptualization, investigation, writing – review and editing. DM: Data curation, formal analysis; BA: Data curation, formal analysis; AO-T: Resources, review and editing. CT: Conceptualization, supervision, writing – review and editing. PD-R: Conceptualization, project administration, visualization, supervision, writing – review and editing. All authors contributed to the article and approved the submitted version.

## Funding

This work was supported by the Spanish Ministry of Science, Innovation and Universities (projects AGL2017-85494-C2-1-R, AGL2017-85494-C2-2-R and PID2020-113268RB-I00) and by the Comunidad de Madrid (grants 2016-T1/BIO-1672 and 2018-T2/BIO-10874). The research was also partially supported by the Strategic Funding UIDB/04423/2020 and UIDP/04423/2020 through national funds provided by the Portuguese Foundation for Science and Technology (FCT) and European Regional Development Fund (ERDF), in the framework of PT2020.

## Conflict of Interest

The authors declare that the research was conducted in the absence of any commercial or financial relationships that could be construed as a potential conflict of interest.

## Publisher’s Note

All claims expressed in this article are solely those of the authors and do not necessarily represent those of their affiliated organizations, or those of the publisher, the editors and the reviewers. Any product that may be evaluated in this article, or claim that may be made by its manufacturer, is not guaranteed or endorsed by the publisher.
