# Supplementary material for: Bacillus subtilis Expressing the Infectious Pancreatic Necrosis Virus VP2 Protein Retains Its Immunostimulatory Properties and Induces a Specific Antibody Response
Source: Front Immunol. 2022 Jun 1;13:888311. doi: 10.3389/fimmu.2022.888311 (PMC9198257; doi:10.3389/fimmu.2022.888311)
Supplement: Supplementary file 1 [file Presentation_1.pptx]

## Slide 1
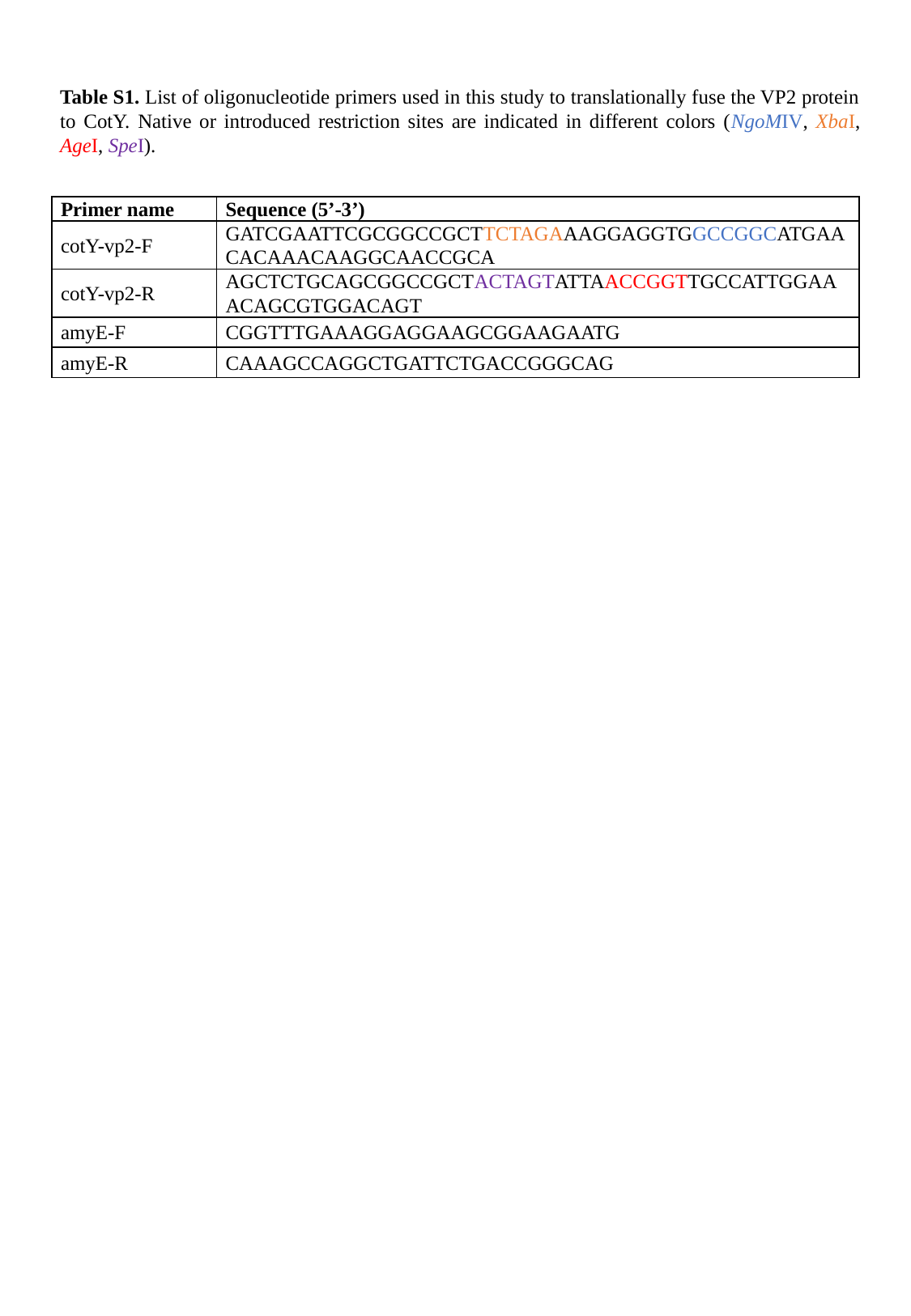

Table S1. List of oligonucleotide primers used in this study to translationally fuse the VP2 protein to CotY. Native or introduced restriction sites are indicated in different colors (NgoMIV, XbaI, AgeI, SpeI).
| Primer name | Sequence (5’-3’) |
| --- | --- |
| cotY-vp2-F | GATCGAATTCGCGGCCGCTTCTAGAAAGGAGGTGGCCGGCATGAACACAAACAAGGCAACCGCA |
| cotY-vp2-R | AGCTCTGCAGCGGCCGCTACTAGTATTAACCGGTTGCCATTGGAAACAGCGTGGACAGT |
| amyE-F | CGGTTTGAAAGGAGGAAGCGGAAGAATG |
| amyE-R | CAAAGCCAGGCTGATTCTGACCGGGCAG |

## Slide 2
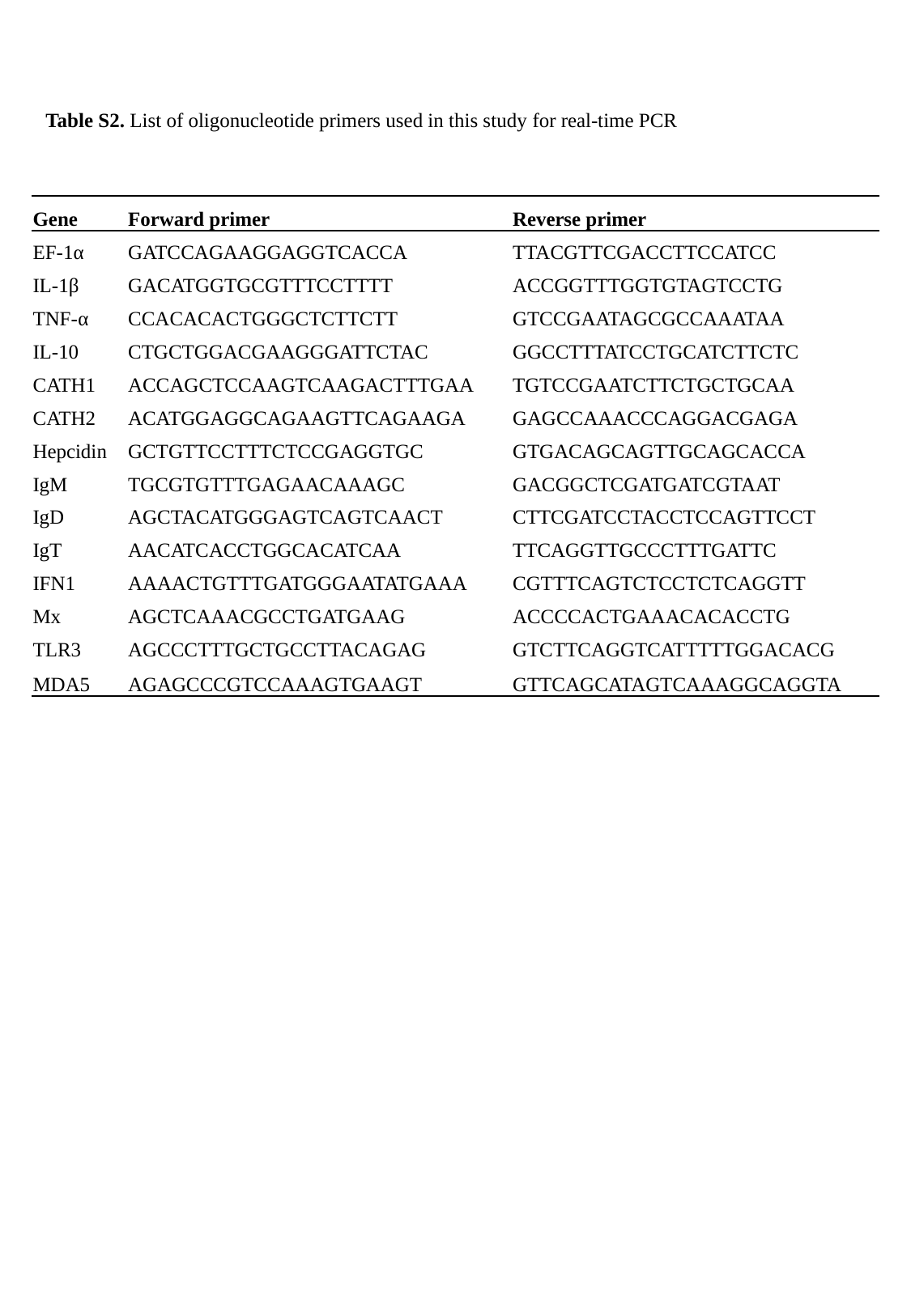

Table S2. List of oligonucleotide primers used in this study for real-time PCR
| Gene | Forward primer | Reverse primer |
| --- | --- | --- |
| EF-1α | GATCCAGAAGGAGGTCACCA | TTACGTTCGACCTTCCATCC |
| IL-1β | GACATGGTGCGTTTCCTTTT | ACCGGTTTGGTGTAGTCCTG |
| TNF-α | CCACACACTGGGCTCTTCTT | GTCCGAATAGCGCCAAATAA |
| IL-10 | CTGCTGGACGAAGGGATTCTAC | GGCCTTTATCCTGCATCTTCTC |
| CATH1 | ACCAGCTCCAAGTCAAGACTTTGAA | TGTCCGAATCTTCTGCTGCAA |
| CATH2 | ACATGGAGGCAGAAGTTCAGAAGA | GAGCCAAACCCAGGACGAGA |
| Hepcidin | GCTGTTCCTTTCTCCGAGGTGC | GTGACAGCAGTTGCAGCACCA |
| IgM | TGCGTGTTTGAGAACAAAGC | GACGGCTCGATGATCGTAAT |
| IgD | AGCTACATGGGAGTCAGTCAACT | CTTCGATCCTACCTCCAGTTCCT |
| IgT | AACATCACCTGGCACATCAA | TTCAGGTTGCCCTTTGATTC |
| IFN1 | AAAACTGTTTGATGGGAATATGAAA | CGTTTCAGTCTCCTCTCAGGTT |
| Mx | AGCTCAAACGCCTGATGAAG | ACCCCACTGAAACACACCTG |
| TLR3 | AGCCCTTTGCTGCCTTACAGAG | GTCTTCAGGTCATTTTTGGACACG |
| MDA5 | AGAGCCCGTCCAAAGTGAAGT | GTTCAGCATAGTCAAAGGCAGGTA |

## Slide 3
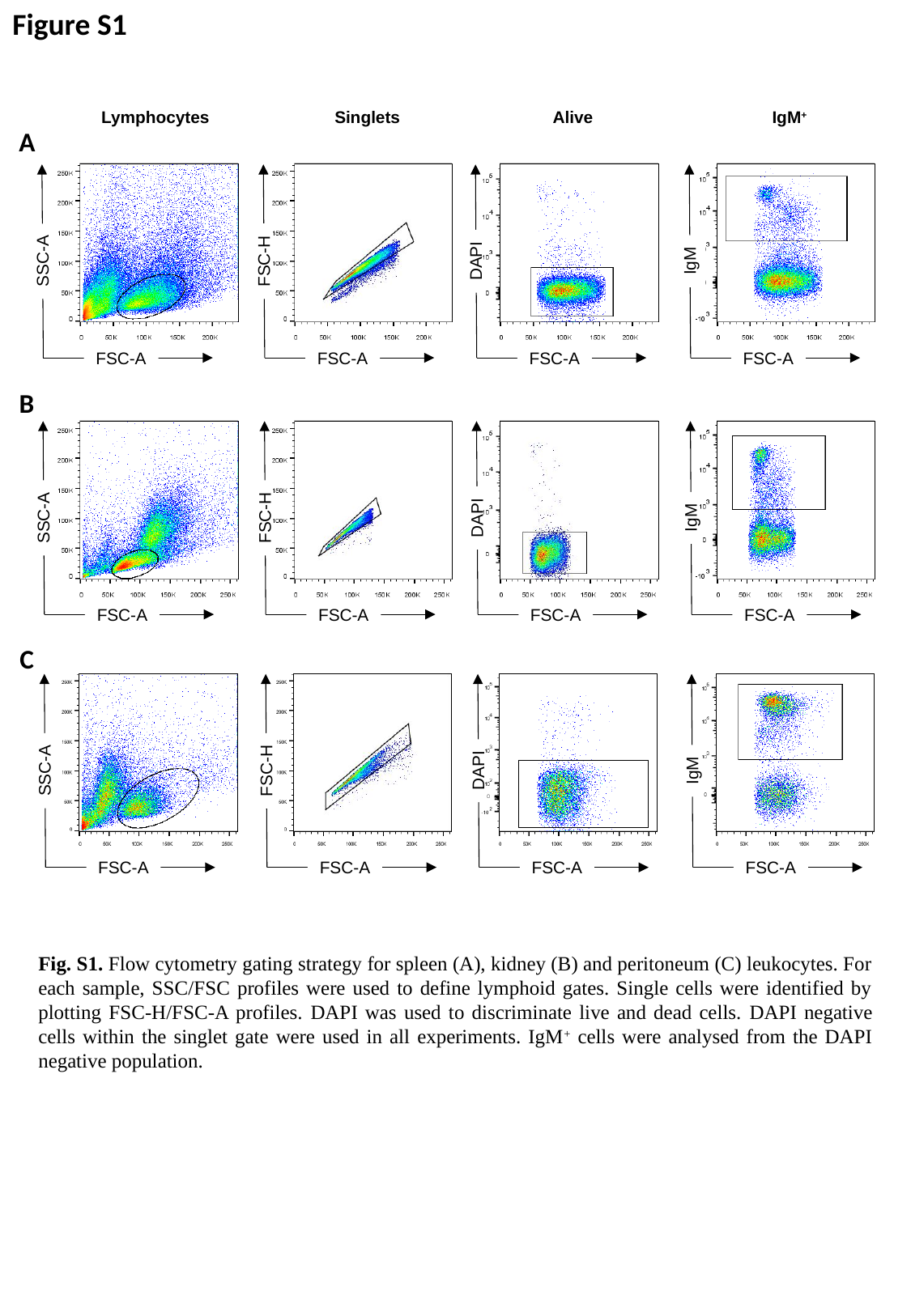

Figure S1
Lymphocytes
Singlets
Alive
IgM+
A
SSC-A
FSC-A
FSC-H
FSC-A
DAPI
FSC-A
IgM
FSC-A
B
SSC-A
FSC-A
FSC-H
FSC-A
DAPI
FSC-A
IgM
FSC-A
C
SSC-A
FSC-A
FSC-H
FSC-A
DAPI
FSC-A
IgM
FSC-A
Fig. S1. Flow cytometry gating strategy for spleen (A), kidney (B) and peritoneum (C) leukocytes. For each sample, SSC/FSC profiles were used to define lymphoid gates. Single cells were identified by plotting FSC-H/FSC-A profiles. DAPI was used to discriminate live and dead cells. DAPI negative cells within the singlet gate were used in all experiments. IgM+ cells were analysed from the DAPI negative population.

## Slide 4
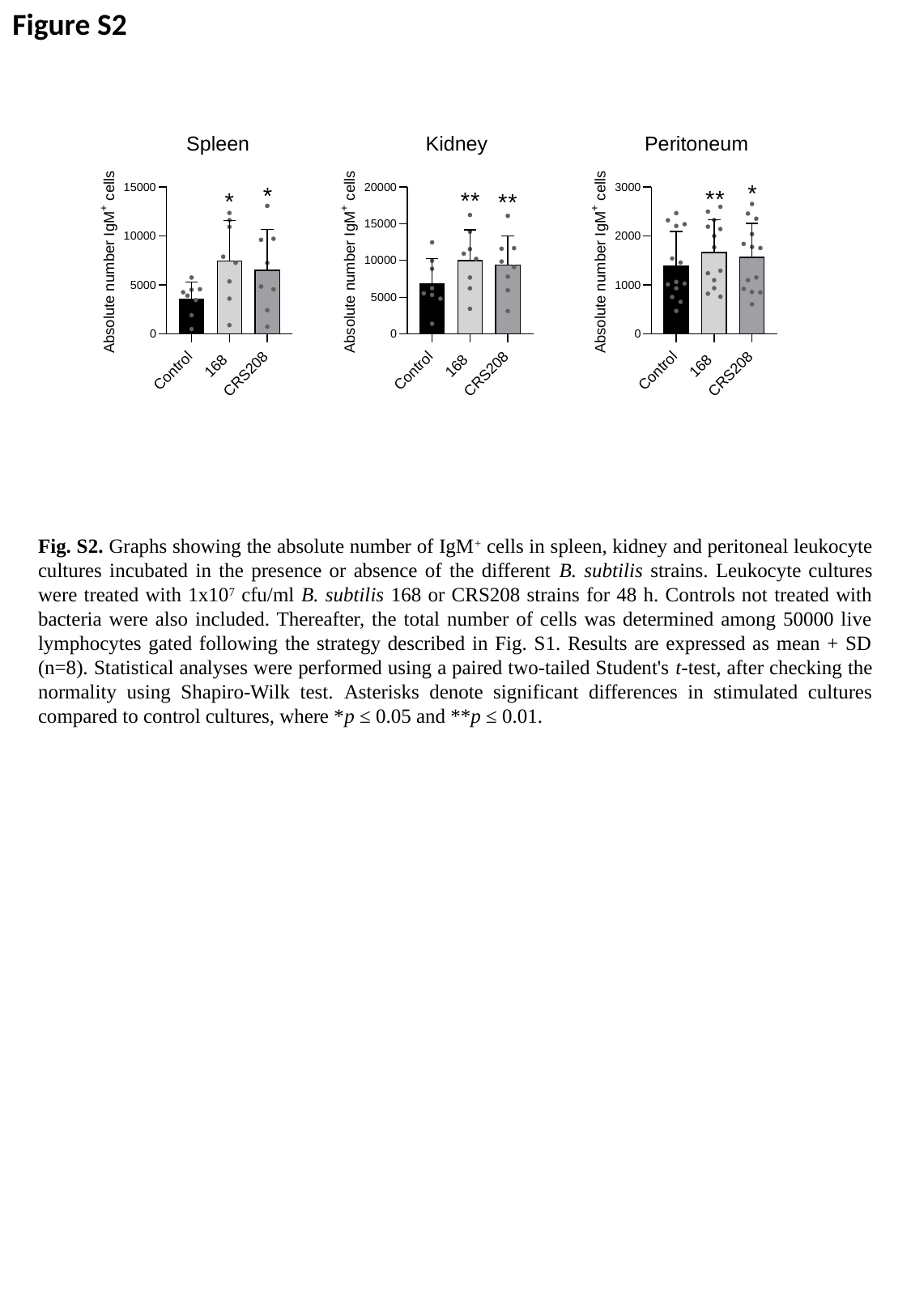

Figure S2
Spleen
Kidney
Peritoneum
Fig. S2. Graphs showing the absolute number of IgM+ cells in spleen, kidney and peritoneal leukocyte cultures incubated in the presence or absence of the different B. subtilis strains. Leukocyte cultures were treated with 1x10^7^ cfu/ml B. subtilis 168 or CRS208 strains for 48 h. Controls not treated with bacteria were also included. Thereafter, the total number of cells was determined among 50000 live lymphocytes gated following the strategy described in Fig. S1. Results are expressed as mean + SD (n=8). Statistical analyses were performed using a paired two-tailed Student's t-test, after checking the normality using Shapiro-Wilk test. Asterisks denote significant differences in stimulated cultures compared to control cultures, where *p ≤ 0.05 and **p ≤ 0.01.
